# Wear Resistance Mechanism of Alumina Ceramics Containing Gd_2_O_3_

**DOI:** 10.3390/ma11102054

**Published:** 2018-10-21

**Authors:** Tingting Wu, Jianxiu Su, Yongfeng Li, Hongyuan Zhao, Yaqi Zhang, Mingming Zhang, Bolin Wu

**Affiliations:** 1 Research Branch of Advanced Materials & Green Energy, School of Mechanical and Electrical Engineering, Henan Institute of Science and Technology, Xinxiang 453000, China; jxsu2003@hist.edu.cn (J.S.); yongfengli121@outlook.com (Y.L.); hongyuanzhao@126.com (H.Z.); zhangyaqi_1988@163.com (Y.Z.); zmmhqm@163.com (M.Z.); 2 College of Material Science and Engineering, Guilin University of Technology, Guilin 541004, China

**Keywords:** alumina ceramics, Gd_2_O_3_, wear resistance, mechanism

## Abstract

Excellent wear resistance of alumina ceramics is a desirable quality for many products. The purpose of this work was to improve the wear resistance of 99% alumina ceramics in an Al_2_O_3_–Gd_2_O_3_–SiO_2_–CaO–MgO (AGSCM) system. The content of Gd_2_O_3_ varied from 0.01% to 1%. A test of wear rate was performed in a ball milling apparatus in a water environment according to the Chinese industry standard. The compositions and microstructure of this material, as well as the effect of bulk density on wear rate, were studied. The effect of Gd_2_O_3_ on phases, grain growth mode, and grain boundary cohesion was investigated. It was found that Gd_2_O_3_ could refine grain size, form compressive stress of the grain boundary, and promote the crystallization of CaAl_12_O_19_. The wear rate of this material was as low as 0.00052‰ (the Chinese industry standard wear rate is ≤0.15‰). The mechanisms for wear resistance of AGSCM ceramics were also determined.

## 1. Introduction

Wear can result in economic waste and safety risks due to maintenance or replacement costs and loss of production or availability [1,2] in equipment such as valves, bearings, cutting tools, sandblasting nozzles, artificial joints, grinding media, etc. Alumina ceramics are attractive for many industrial applications due to their excellent properties and relatively low cost of manufacture [3,4].

There has been much research on the wear resistance of ceramics. Grain size is deemed to be one of the most important factors in determining wear rate of ceramics. Doğan [5] discovered that grain size must be tiny for optimal wear resistance regardless of the content of Al_2_O_3_. Meng et al. [6] prepared two different grain-size alumina materials by spark plasma sintering technique to study their tribological behavior. The result indicated that the wear loss volume of the sample with finer grain was only half that of the sample with coarser grain. However, Esposito [4] thought that the composition of the second phase and the content of the glass phase were fundamental causes in determining the nature of wear. David [7] confirmed that alumina ceramics with glass phase at the grain boundary were much more resistant to fatigue wear than alumina ceramics containing no or very little glass phase. Goswami et al. [8] also put forward a point that the wear resistance of liquid-phase-sintered alumina ceramics was better than highly pure polycrystalline alumina ceramics. Zhou et al. [9] reported that La_2_O_3_/Y_2_O_3_ improved the wear resistance and toughness of ceramics through the “pull-out” effect. However, Lee et al. [10] pointed out that the effect of toughness on wear resistance was insignificant. They noted that the wear resistance of material decreased rather than increased with an increase in toughness. There are therefore different views on the influence factors of wear resistance.

The development of science and technology has not only brought a higher requirement for wear resistance but also demanded higher purity for materials, such as insulators used for high-pressure and extra-high voltage systems, electric components with low dielectric loss and good surface finish, ceramic tubes used for lamps, precision components in aerospace domain, etc. The impurity content is the key issue in preparation of high-purity alumina ceramics. Ball milling is an important link of production in many industrial sectors, such as silicate, powder metallurgy, food, medicine, and other industries [11]. Grinding media are indispensable materials for ball milling. However, they are also one of the main pollution sources. Therefore, the quality of alumina grinding media is an important factor that affects the quality of products. For example, the raw materials of 99% alumina ceramics must be milled using 99% alumina grinding media with outstanding wear resistance, which can reduce the pollution from abrasion. However, the sintering temperature of 99% alumina ceramics is too high, and its glass phase content is too little. Therefore, the grains always become coarse and uneven, leading to weak wear resistance. The need to improve the wear resistance of high alumina ceramics is thus imperative.

Most studies in this field have proven that the wear resistance of alumina ceramics not only has a strong dependence on the microstructure [12,13] but is also relevant to the chemical composition of the ceramics [14,15]. Rare earths can greatly improve the quality and properties of materials. They are known as “the vitamin of modern industry”. A lot of researches have reported that rare-earth oxides can improve density, strength, hardness, thermal stability, creep resistance of materials, etc. [16,17,18,19,20,21,22]. However, the effect of rare-earth oxides on wear resistance mechanism has not been systematically investigated. The application of Gd_2_O_3_ has made a satisfactory progress in recent years [23,24,25], but few investigations on the wear-resistant mechanism of materials have been reported. The Hund rule says that the electronic shell is more stable when it achieves the states of completely empty, completely full, or half-full. The ground state electrons of La^3+^, Gd^3+^, and Lu^3+^ are [Xe]4f^0^, [Xe]4f^7^, and [Xe]4f^14^, respectively. La_2_O_3_ and Lu_2_O_3_ can improve the wear resistance of alumina ceramics [26,27]. However, whether Gd_2_O_3_ can also improve the wear resistance of alumina ceramics is unclear. Many experiments have confirmed that the sintering temperature of CaO–MgO–Al_2_O_3_–SiO_2_ system is low because the liquid phase appears at low temperature. In this study, we reveal the mechanism of Gd_2_O_3_ that influences the wear resistance of high alumina ceramics in CaO–MgO–Al_2_O_3_–SiO_2_ system by studying their density, wear rate, phase composition, and microstructure.

## 2. Materials and Methods

High alumina ceramics were prepared in an Al_2_O_3_–Gd_2_O_3_–SiO_2_–CaO–MgO (AGSCM) system. Samples were prepared using a commercial alumina powder (>99.8% purity; a mean particle size of 0.65 μm; chemical composition: Na_2_O: 0.1%, Fe_2_O_3_: 0.03%, SiO_2_: 0.05%, TiO_2_: 0.005, CaO: 0.05%, MgO: 0.1%). The alumina powder was mixed with magnesia, calcium carbonate, silicon dioxide, and gadolinium oxide. The mass proportions of raw materials were weighed (Al_2_O_3_ and Gd_2_O_3_: 99 wt%; CaO: 0.33 wt%; MgO: 0.33 wt%; SiO_2_: 0.34 wt%). When the Gd_2_O_3_ contents were 0 wt%, 0.01 wt%, 0.1 wt%, and 1 wt%, the samples were referred to as Sample 1, 2, 3, and 4, respectively. The powders were mechanically milled for 24 h using a ball mill and then shaped to obtain green compacts (sphere of 30mm in diameter) by cold isostatic pressing at 100 MPa. They were sintered at 1475 °C for 1 h in air. After these processes, bulk density and wear rate were tested. Bulk density was measured by the Archimedes method. Wear rate was tested in accordance with Chinese Building Materials Industry Standard JC/T 848.1-2010 (alumina grinding ball). The process was as follows: We first weighed a sample (*M*_1_) and measured its diameter (*D_x_*). A 1 kg sample and 1 L water were put into a polyurethane pot and then milled for 24 h. The speed of the ball mill was 80 rpm. The sample was then dried and weighed again (*M*_2_). The process was repeated for each sample. The wear rate was calculated using the following equation:

*W* = K*D* (*M*_1_ − *M*_2_)/*M*_1_ (1)

where *W* is wear rate (‰); K is a constant (4.17 × 10^−4^ mm^−1^); *D* is the mean diameter (mm) of samples; *M*_1_ is the weight of sample before wear (g); *M*_2_ is the weight of sample after wear (g). Based on the industry standard, the wear rate of alumina grinding ball (alumina content of about 99 wt%) should be less than 0.15‰.

Phase compositions of samples were identified by comparing the X-ray powder diffraction (Cu K_α_) patterns with the standard chart. The test was carried out in an “X’Pert PRO” multipurpose X-ray diffractometer (PANalytical B.V., Almelo, Netherlands) with 40 kV voltage and 40 mA current. X-ray patterns were taken by measuring 2θ from 5° to 90° at a step size of 0.02° and a dwell time of 5 s per step. The results of the powder diffraction patterns were analyzed with X’Pert High Score Plus software. The microstructures of the sintered samples were characterized using field emission scanning electron microscope (FESEM, S-4800, Hitachi, Japan) equipped with energy dispersive spectroscopy (EDS). Thermal expansion of the glass phase was tested by dilatometer (DIL402C, NETZSCH Group). Cylindrical samples (L = 26 mm, Φ = 5 mm) were prepared according to the blending ratio of flux in the alumina ceramics. The sample was then put into the dilatometer. One end of the sample was anchored, and the other end of the sample contacted with the ejector rod. The sample, support, and ejector rod were raised to a high temperature at the same time. Their difference values were conveyed by the ejector rod. Due to the effect of equipment parts, the calculation of thermal expansion coefficient needed to add a modified value.

## 3. Results

### 3.1. Wear Rate and Bulk Density

**Figure 1:**
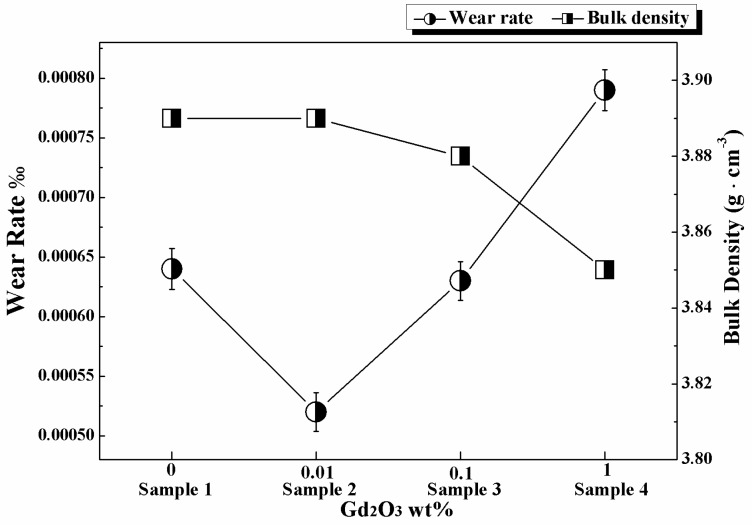
Relationship between Gd_2_O_3_ content, wear rate, and bulk density.

Bulk density is a reliable index for reflecting the densification of ceramic materials. Wear rate is an index to evaluate the wear resistance of ceramics. According to Chinese Building Materials Industry Standard, wear rate of 99% alumina grinding ball should be less than 0.15‰, and its bulk density should be more than 3.85 g/cm^3^. The wear rates and bulk densities of samples are shown in Figure 1.

The density curve shows that the bulk density decreased with the increase in Gd_2_O_3_ content. Sample 2 contained 0.01% Gd_2_O_3_, and its density was 3.89 g/cm^3^, which was the same as Sample 1 without Gd_2_O_3_. This meant that a trace amount of Gd_2_O_3_ did not affect the density of alumina ceramics too much. When Gd_2_O_3_ content reached 0.1 wt%, the density of Sample 3 declined notably. When Gd_2_O_3_ content was 1 wt%, the density of Sample 4 was the minimum (3.85 g/cm^3^). The results indicated that adding Gd_2_O_3_ was detrimental to the bulk density of alumina ceramics.

The curve of wear rate varied parabolically with the increase in Gd_2_O_3_ content. The wear rate of Sample 2 containing 0.01% Gd_2_O_3_ fell sharply to 0.00052‰, which reached the minimum. Under equivalent conditions, the wear rate of a product with good wear resistance on the market is 0.125‰. The wear resistance of Sample 2 was better than that of Sample 1 (0.00064‰) and had been enhanced nearly 200 times over the product. However, the wear rates of Samples 3 and 4 rose rapidly and were higher than that of Sample 1. The results indicated that trace amounts of Gd_2_O_3_ could effectively improve the wear resistance of alumina ceramics. The appropriate quantity of Gd_2_O_3_ was 0.01 wt%, and excessive Gd_2_O_3_ resulted in weak wear resistance and low density of alumina ceramics.

### 3.2. Phase Composition

**Figure 2:**
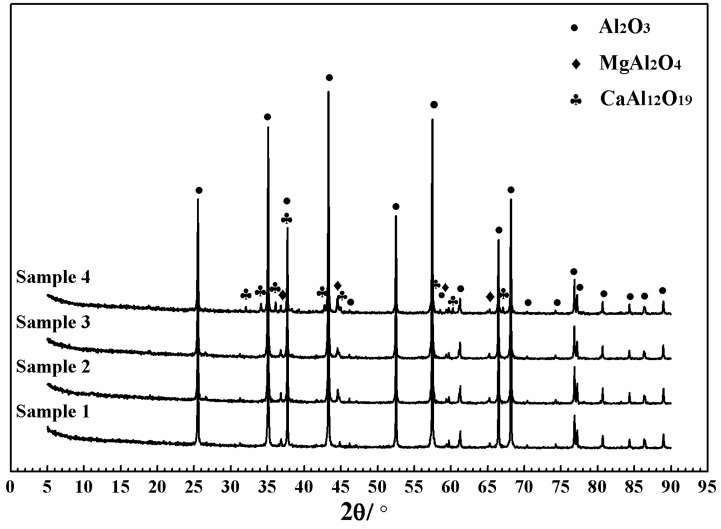
Powder X-ray diffraction patterns of samples with different Gd_2_O_3_ concentrations (Sample 1: 0 wt%; Sample 2: 0.01 wt%; Sample 3: 0.1 wt%; Sample 4: 1 wt%).

Phase compositions of the samples with different Gd_2_O_3_ contents were measured and analyzed. The X-ray diffraction patterns of samples are shown in Figure 2. The diffraction peaks of Al_2_O_3_ and MgAl_2_O_4_ were identified in all four samples. With the increase in Gd_2_O_3_ content, a new phase (CaAl_12_O_19_) was observed in Sample 4. This indicated that Gd_2_O_3_ could promote CaAl_12_O_19_ generation; however, the crystalline phase containing Gd was undetected.

**Figure 3:**
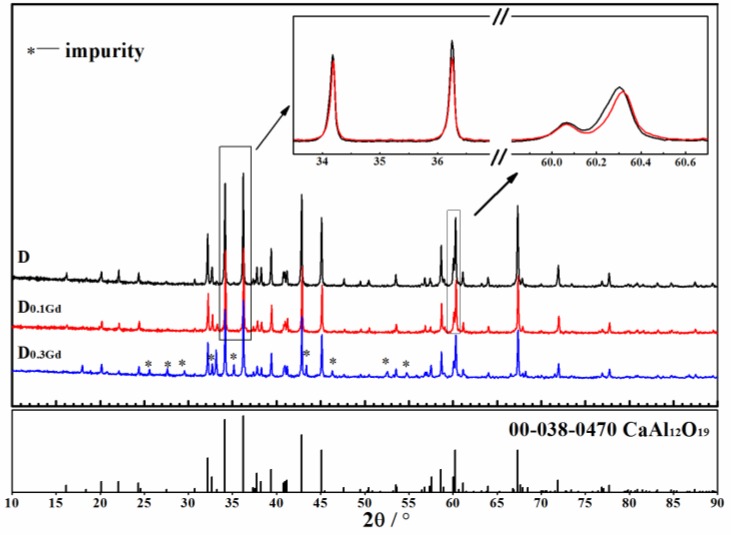
X-ray diffraction patterns of (Gd_x_,Ca_1−x_)Al_y_O_19._

The ionic radius of Ca is 0.099 nm, which is approximately that of Gd (0.097 nm) [28]. Major factors that affect the solid solution to form include ionic radius and valence states. The difference between the two ionic radii is less than 15%, which is a favorable condition for forming a substitutional solid solution. When the valence of elements is different, compounds can keep electric neutrality by changing the structure [29]. Therefore, we deduced that Gd^3+^ replaced Ca^2+^ to form solid solutions with CaAl_12_O_19_. In order to reveal where Gd_2_O_3_ went and the generation causes of CaAl_12_O_19_, the solid solution experiment of Gd_2_O_3_ and CaAl_12_O_19_ was designed. CaAl_12_O_19_ was synthesized (Sample D). Then, 10 mol% or 30 mol% of Gd was used to replace Ca and Samples D_x_-(Gd_x_,Ca_1−x_)Al_y_O_19_ were prepared (Table 1). The samples were characterized by XRD. The results are shown in Figure 3.

The diffraction peaks of Sample D and D_0.1Gd_ matched well with the PDF (Powder Diffraction File) card of CaAl_12_O_19_. There was no impurity in them. However, there were some impurities when the content of Gd reached 30 mol% (Sample D_0.3Gd_). This indicated that Sample D_0.3Gd_ was not a pure substance. In order to calculate lattice parameters, slow scan was taken by measuring 2θ from 33.5° to 37.5° and from 59.8° to 60.6° at a step size of 0.017° and a dwell time of 122 s per step. The result showed that the diffraction peaks of D_0.1Gd_ shifted slightly in varying degrees. Because the ionic radius of Gd is smaller than that of Ca, the crystalline interplanar spacing should decrease when Gd^3+^ replaces Ca^2+^ in CaAl_12_O_19_. It was derived that θ should be increased according to the Bragg equation (2dsinθ = λ), so diffraction peaks of D_0.1Gd_ would shift slightly to the high-angle region. The lattice parameters of D and D_0.1Gd_ were calculated by MDI Jade 6.0 software. The results are shown in Table 2.

CaAl_12_O_19_ belongs to the hexagonal system, so its lattice parameters are different (a = b ≠ c). Grain growth of CaAl_12_O_19_ is anisotropic. Crystal nucleus preferentially grows along the basal plane to form a plate or tabular crystal [30,31]. As can be seen in Table 2, the lattice parameter a (b) of Sample D_0.1Gd_ was longer than that of Sample D, and the lattice parameter c of Sample D_0.1Gd_ was shorter than that of Sample D. However, compared with Sample D, lattice volume of Sample D_0.1Gd_ decreased. This conforms to the fact that the ionic radius of Gd is smaller than that of Ca. The results demonstrated that Gd_2_O_3_ could promote the formation of CaAl_12_O_19_ by forming solid solutions. In the ceramics containing Gd_2_O_3_, the CaAl_12_O_19_ phase was the solid solution of Gd_2_O_3_ and CaAl_12_O_19_ (in Figure 2).

### 3.3. Microstructure

**Figure 4:**
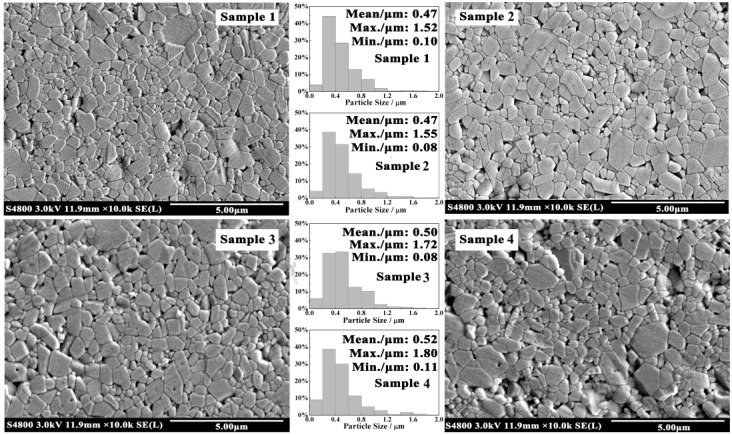
Field emission scanning electron microscope (FESEM) photographs of samples. The wear resistance of Sample 2 (0.01 wt% Gd_2_O_3_) is the best; the wear resistance of Samples 3 and 4 are worse than that of Sample 1 without Gd_2_O_3_.

Four samples were cut and polished for FESEM testing. The microstructure of the cross section is shown in Figure 4. With the increase in Gd_2_O_3_ content, the grain size of the sample took on a trend of polarization: The size of small grains decreased and their quantity increased; however, the size of big grains increased and the average grain size increased.

A comparison between the four samples showed that the average grain sizes of Samples 1 and 2 were the smallest. A bar graph of grain size distribution showed that the percentage of small grains whose size was less than 0.2 μm was about 5%. The small grain size of Sample 2 (0.08 μm) was smaller than that of Sample 1 (0.1 μm), but the size of big grain increased. Even so, the average grain size of Sample 2 was the same as that of Sample 1. Besides, the microstructures of both were similar. There were pores in the corner of grains, which were left over from the intergranular voids in starting materials briquetting. Therefore, the pores were granular inclusions at the grain boundary. The pores moved together with interface to gradually gather at the corners of grains [29]. The difference between Sample 1 and Sample 2 was that the pores of the grain interior decreased in Sample 2. Pores can be left behind by the moving interface to form pores of the grain interior [29]. The results indicated that Gd_2_O_3_ could reduce the migration rate of grain boundary to provide ample time for removing pores.

The smallest grain size of Sample 3 (Gd_2_O_3_: 0.1 wt%) was also 0.08 μm, which was the same as Sample 2. However, the number of small grains increased to 6% in Sample 3. Dopants can alter the boundary, lattice, and surface diffusivities, leading to a change in boundary [32,33]. Large rare-earth cations segregate at the grain boundaries, which can create drag forces on the boundary motion and block the diffusion of other ions along grain boundaries. This results in reducing grain-boundary diffusivity and inhibiting grain growth. By comparing the size and quantity of small grains, it was discovered that moderate amounts of Gd_2_O_3_ could make the grains finer and inhibit grain growth.

The smallest grain size, the biggest grain size, and the average grain size of Sample 4 were all bigger than the other samples. In Sample 4, the content of Gd_2_O_3_ was the highest. When the content of rare-earth oxide is too much, the microregion liquid can be formed easily because of the impurities (K^+^, Na^+^) in the raw material and rare-earth oxide. Pillai et al. [33] reported that when the impurity content at the grain boundary attained a critical level, liquid phases of thermodynamically stable thickness could be formed and could induce a sudden increase in the mobility of grain boundaries that were wetted by the liquid film at higher temperature, thus resulting in grain growth. Besides, the reduction of grain size resulted in an increased number of grain boundaries. The large rare-earth ions were not enough to cover all grain boundaries, causing individual abnormal grain growth [34]. Ucar et al. [3] believed that the large grain size caused large particles coming off during the wear process. In addition to the impact on grain size, Gd_2_O_3_ also affected the density of alumina ceramics. The microstructures of Sample 1, 2, and 3 were not changed very much; they were similar in pore size and pore quantity. However, the pore size and pore quantity of Sample 4 increased markedly, and its density declined dramatically. The result was consistent with the bulk density (Figure 1). Poor density is bad for wear resistance of materials.

**Figure 5:**
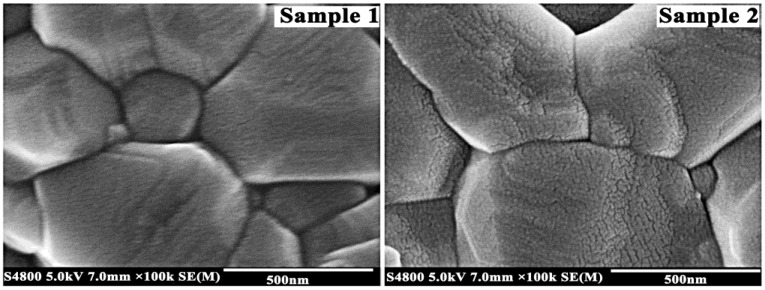
FESEM photographs of microstructure between grains of Samples 1 and 2.

The results indicated that trace addition of Gd_2_O_3_ could make the grains finer and inhibit grain growth by slowing down the rate of grain boundary. Therefore, the performance of ceramics was improved. In order to uncover how to improve the wear resistance of alumina ceramics by adding trace amounts of Gd_2_O_3_, microstructures between grains of Samples 1 and 2 were studied (Figure 5).

**Figure 6:**
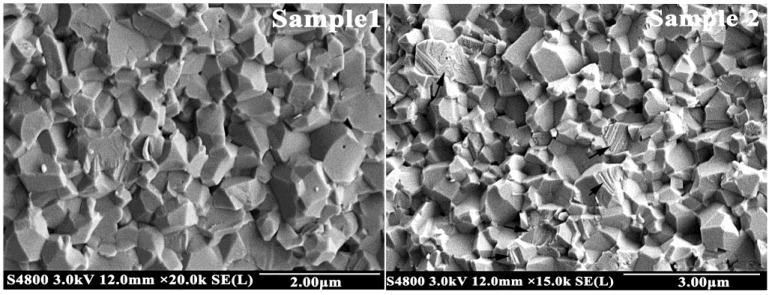
Fracture surfaces of Samples 1 and 2.

In Sample 1, the length–width ratio of grains was small and the grain boundary was broad. The voids were big at the intersectional parts of three grains. In Sample 2, preferred orientation growth of grains was obvious. The grain surface presented squeezing growing lines at the intersectional parts of three grains. On the one hand, squeezing growth among grains plays a role of inhibiting grain growth; on the other hand, compressive stress of the grain boundary can improve the toughness of the materials and consume the foreign energy, thus improving the brittleness of ceramic materials [35]. The feature of Sample 2 was compressive stress of the grain boundary. Its grain boundary was narrower than Sample 1. Generally, the array of grains is disorderly at the grain boundary, which leads to many defects and loose structure. Therefore, grain boundaries are vulnerable to damage. Narrow grain boundary not only reduces the defects but also shortens the distance to improve binding force between adjacent grains. This was reflected in the fracture morphology (Figure 6).

The relationship between the fracture morphology of materials and mechanical property was linear: When the grain size was similar, the larger the structural fluctuation of fracture, the more complex was the crack propagation path. More energy was consumed during the fracturing process. This meant that the materials had high wear resistance. Otherwise, the flatter the structural fluctuation of fracture, the worse was its property [36]. As can be seen in Figure 6, the grain size of Sample 1 was very close to that of Sample 2. Fracture mode included intergranular fracture and transgranular fracture. Fracture paths of Sample 1 were straight, and the cleavage step of grain was observed at transgranular fracture (arrow), which displayed the characteristic of cleavage fracture; its fracture morphology was flat. The fracture of Sample 2 was uneven and very crude, indicating that the fracture paths of Sample 2 were zigzag. Some tearing ridges appeared in transcrystalline fracture of grain. The winding path and tearing ridge (as indicated by the black arrows) reflected that more fracture energy was consumed in the sample containing rare-earth oxide. Therefore, it was proven that the binding forces between grains were very strong.

### 3.4. Effect of Gd_2_O_3_ on the Thermal Expansion of Glass Phase

**Figure 7:**
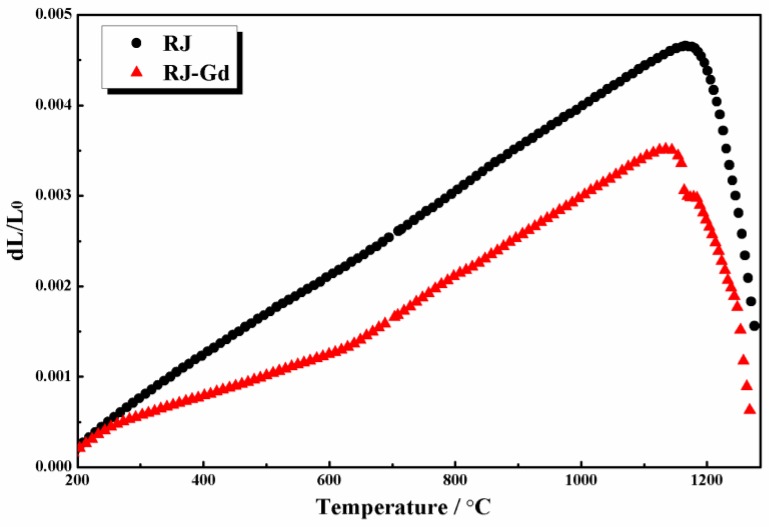
Thermal expansion curves of fluxes.

In ceramics, rare-earth ions are mainly found in the crystalline phase and the glass phase. The difference in thermal expansion coefficients between alumina and glass phase has a great impact on the wear resistance of alumina ceramics. The glass phase exists between grains; its thermal expansion behavior mainly affects stress at the grain boundary. The stress is not enough to cause damage (tiny cracks), but the destructive power will be enhanced when it combines with the induced stress of wear [8]. The glass phase expands during the heating process, and its volume contraction is also serious during cooling process. The tensile stress induced by shrinkage deformation is much larger than compressive stress that accumulates during heat-up, which causes high residual tensile stresses at the grain boundary. This is not good for improving wear resistance of materials [29]. In order to research the effect of Gd_2_O_3_ on thermal expansion of glass phase, Sample RJ was prepared according to the blending ratio of flux in alumina ceramics. Sample RJ–Gd was prepared by adding Gd_2_O_3_ into Sample RJ. The content of Gd_2_O_3_ was 0.5wt%. The thermal expansion curves of Sample RJ and RJ–Gd are shown in Figure 7.

Expansion softening point of the two samples was similar. However, the thermal expansion curve of Sample RJ–Gd was below Sample RJ. The expansion quantity of Sample RJ–Gd started to be lower than that of Sample RJ from 300 °C onwards. During cooling, the low expansion quantity of glass had less volume shrinkage deformation, which could decrease residual tensile stress at the grain boundary. Combined with the squeezing growing lines of grain surface (Figure 5) and the thermal expansion curve of Sample RJ–Gd, it is asserted that the tensile stress of glass phase induced by restrained shrinkage deformation was smaller than the accumulated compressive stress on heating. Therefore, the compressive stress was formed at the grain boundary, which was another reason for improving wear resistance of alumina ceramics.

## 4. Discussion

In this study, we discovered that a trace amount of Gd_2_O_3_ could improve the wear resistance of alumina ceramics. The thermal expansion coefficients are different along different crystallographic directions of alumina grains (α_a_ = 8.6 × 10^−6^ °C^−1^ and α_c_ = 9.5 × 10^−6^ °C^−1^), which leads to residual stress in ceramics. The big and inhomogeneous residual stress can cause self-forming microcracks inside materials [37]. However, small grain size and narrow distribution can reduce the tensile stresses caused by the anisotropy of thermal expansion coefficients among different grains, thus enhancing the wear resistance of materials [38]. A number of researchers [5,8,39] have noted that ceramics with smaller crystal size demonstrate a higher level of physical properties. Evans had pointed that the brittleness of ceramics was the major cause of wear [40,41]. The manifestation of wear is fracture. Compared with coarser-grained ceramics, an increased amount of grain boundary leads to a zigzag fracture path in fine-grained ceramics. The zigzag fracture path is not conducive to the propagation of cracks, which effectively blocks fracture of ceramics and improves wear resistance. Therefore, grain size has been considered the main factor in determining wear resistance—a view that is consistent with the experimental results.

Our research found that trace amounts of Gd_2_O_3_ could inhibit grain growth. Rare-earth elements belong to surface-active material, but their solubility in alumina is extremely low; they segregate easily at the grain boundary. On the one hand, they can fill in the defects on the grain surface to reduce the surface energy, inhibiting grain growth [36]. On the other hand, rare-earth ions block the diffusion of other ions along grain boundaries because of their bigger radii [42,43,44] and reduce the grain boundary diffusivity (Figure 4). However, a decreased grain size results in an increased number of grain boundaries. The rare-earth ions are not enough to cover all grain boundaries, causing individual abnormal grain growth. This is the reason the maximum grain size of Sample 2 was bigger than that of Sample 1, although it did not affect the average grain size of the two samples.

In the same average grain size, we found that microstructure played a decisive role in wear resistance of materials. The ratio of transgranular fracture increased in the sample containing trace amounts of Gd_2_O_3_. Many tearing ridges indicated that more energy was consumed during the fracture process of Sample 2 (Figure 6). It was also demonstrated that adding trace amounts of Gd_2_O_3_ could enhance bonding strength between adjacent grains by narrowing the grain boundary (Figure 5). Besides, squeezing growing lines verified that compressive stress existed in the sample containing Gd_2_O_3_. As far as theory is concerned, if stresses created by the boundary glass are tensile, the ceramics will be more susceptible to wear, whereas if these stresses are compressive, the wear resistance of material will be enhanced [38]. Gd_2_O_3_ could dramatically reduce the thermal expansion quantity to decrease residual tensile stress caused by volume shrinkage deformation of glass phase during cooling. Besides, Gd^3+^ could replace Ca^2+^ in CaAl_12_O_19_ to form a solid solution (Figure 3). This means that adding Gd_2_O_3_ had a certain purifying effect on the grain boundary. Cynthia discovered that the thermal expansion coefficient of high CaO glass (α = 9.5 × 10^−6^ °C^−1^) was higher than that of alumina (8.6 × 10^−6^ °C^−1^) [45]. Thus, the interface between glass and Al_2_O_3_ grains could form tensile stresses, resulting in a weaker boundary. The tensile stress at the grain boundary could be effectively mitigated by reducing calcium ingredient of glass phase. In addition, the thermal expansion coefficient of CaAl_12_O_19_ is 8.0 × 10^−6^ °C^−1^ [30], which is lower than that of Al_2_O_3_, and both could easily form compressive stress at grain boundary. All of these factors came together to improve the wear resistance of alumina ceramics.

## 5. Conclusions

In this study, we found that adding trace amounts of Gd_2_O_3_ could improve the wear resistance of high-alumina ceramics. The wear rate was as low as 0.00052‰, and the optimum quantity was less than 0.01 wt%. The effect of Gd_2_O_3_ on changing residual stress at the grain boundary was significant. On the one hand, trace amounts of Gd_2_O_3_ could refine grain without impacting density of ceramics, which was useful for decreasing the residual stress caused by anisotropy thermal expansion of alumina grain. On the other hand, Gd_2_O_3_ could significantly reduce thermal expansion quantity of glass. Gd_2_O_3_ also promoted CaAl_12_O_19_ with lower thermal expansion coefficient to form via solid solution. The various factors came together to form compressive stress at the grain boundary. Combined with the effect of purifying and narrowing grain boundary, enhancing the binding force between adjacent grains improved wear resistance. However, the results showed that excessive addition caused serious grain growth, deteriorating performance.

## Figures and Tables

**Table 1:** Stoichiometric coefficient of (Gd_x_,Ca_1−x_)Al_y_O_19._

No.	Chemical Formula	x	y
D	CaAl_12_O_19_	-	-
D_0.1Gd_	(Gd_0.1_,Ca_0.9_)Al_11.97_O_19_	0.1	11.97
D_0.3Gd_	(Gd_0.3_,Ca_0.7_)Al_11.9_O_19_	0.3	11.9

**Table 2:** Lattice parameters of Sample D and D_0.1Gd._

No.	Stoichiometric Formula	Lattice Parameters		Lattice Volume/V
		a = b	c	
D	CaAl_12_O_19_	5.55272	21.88913	584.48
D_0.1Gd_	(Gd_0.1_,Ca_0.9_)Al_11.97_O_19_	5.5533	21.88022	584.37

## References

[1] Moore M.A. (1978). Abrasive wear. Int. J. Mater. Eng. Appl..

[2] Medvedovski E. (2001). Wear-resistant engineering ceramics. Wear.

[3] Ucar V., Ozel A., Mimaroglu A., Callı I., Gur M. (2001). Influence of SiO_2_ and MnO_2_ additives on the dry friction and wear performance of Al_2_O_3_ ceramic. Mater. Des..

[4] Esposito L., Tucci A. (1997). Microstructural dependence of friction and wear behaviours in low purity alumina ceramics. Wear.

[5] Doğan C., Hawk J. (1999). Role of composition and microstructure in the abrasive wear of high-alumina ceramics. Wear.

[6] Meng F., Zhang X., Zhang Y., Dong Y. (2013). Friction and Wear Behavior of Fine-grained Alumina Ceramic Under Articular Biomimetic Synovial Fluid Conditions. J. Chin. Ceram. Soc..

[7] Dimond C. (1987). The specification and installation of alumina ceramics in industry. Mater. Des..

[8] Goswami A.P., Roy S., Mitra M.K., Das G.C. (2000). Influence of powder, chemistry and intergranular phases on the wear resistance of liquid-phase-sintered Al_2_O_3_. Wear.

[9] Zhou H., Zeng G., Liu J., Zhou Y. (2016). Rare earth oxides La_2_O_3_/Y_2_O_3_-toughened & Reinforced ZTA ceramic and its abrasion resistance. Mater. Rev..

[10] Lee D.G., Lee H., Kim P., Bang K. (2003). Micro-drilling of alumina green bodies with diamond grit abrasive micro-drills. Int. J. Mach. Tools Manuf..

[11] Matsuzaki H., Wako Y., Watanabe M. (2013). Studies on the characteristics of wear in micro-beads, and food-safety of fine particles caused by wear in a micro-bead mill. Powder Technol..

[12] Davidge R., Twigg P., Riley F. (1996). Effects of silicon carbide nano-phase on the wet erosive wear of polycrystalline alumina. J. Eur. Ceram. Soc..

[13] Franco A., Roberts S. (1996). Controlled wet erosive wear of polycrystalline alumina. J. Eur. Ceram. Soc..

[14] Rainforth W.M. (1996). The sliding wear of ceramics. Ceram. Int..

[15] Badmos A.Y., Ivey D.G. (2001). Characterization of structural alumina ceramics used in ballistic armour and wear applications. J. Mater. Sci..

[16] Qiu G., Li X., Qiu T., Zhao H., Yu H., Ma R. (2007). Application of rare earths in advanced ceramic materials. J. Rare Earths.

[17] Fang J., Thompson A.M., Harmer M.P., Chan H.M. (1997). Effect of Yttrium and Lanthanum on the Final-Stage Sintering Behavior of Ultrahigh-Purity Alumina. J. Am. Ceram. Soc..

[18] Yao Y., Qiu T., Jiao B., Shen C. (2004). The Effects of Rare Earth Oxide on the Properties of Alumina Ceramics. Vac. Electron..

[19] West G.D., Perkins J.M., Lewis M.H. (2004). Characterisation of fine-grained oxide ceramics. J. Mater. Sci..

[20] Yang Q., Zeng Z., Xu J., Zhang H., Ding J. (2006). Effect of La_2_O_3_ on Microstructure and Transmittance of Transparent Alumina Ceramics. J. Rare Earths.

[21] Loudjani M., Huntz A., Cortes R. (1993). Influence of yttrium on microstructure and point defects in α-Al_2_O_3_ in relation to oxidation. J. Mater. Sci..

[22] Thompson A.M., Soni K.K., Chan H.M., Harmer M.P., Williams D.B., Chabala J.M., Levi-Setti R. (1997). Dopant distributions in rare earth doped alumina. J. Am. Ceram. Soc..

[23] Guo H., Dong N., Yin M., Zhang W., Lou L., Xia S. (2004). Visible Upconversion in Rare Earth Ion-Doped Gd_2_O_3_ Nanocrystals. J. Phys. Chem. B.

[24] Luo N., Yang C., Tian X.M., Xiao J., Liu J., Chen F., Zhang D., Xu D., Zhang Y., Yang G.W. (2014). A general top-down approach to synthesize rare earth doped-Gd_2_O_3_ nanocrystals as dualmodal contrast agents. J. Mater. Chem. B.

[25] Wang H., Huang H., Liang J., Liu J. (2014). Preparation of ZrO_2_/Gd_2_O_3_ composite ceramic materials by coprecipitation method. Ceram. Int..

[26] Wu T.T., Zhou J., Wu B.L., Liu J.C. (2016). Effect of Rare-earth Lu_2_O_3_ on the Wear Resistance of Alumina Ceramics for Grinding Media. Powder Technol..

[27] Wu T.T., Zhou J., Wu B.L., Li W.J.  (2016). Effect of La_2_O_3_ content on wear resistance of alumina ceramics. J. Rare Earths.

[28] Zhou Y. (2004). Science of Ceramic.

[29] Kingery W.D., Bowen H.K., Uhlmann D.R. (2010). Introduction to Ceramics.

[30] Salomão R., Ferreira V.L., Oliveira I.R.D., Souza A.D.V., Correr W.R. (2016). Mechanism of pore generation in calcium hexaluminate (CA6) ceramics formed in situ from calcined alumina and calcium carbonate aggregates. J. Eur. Ceram. Soc..

[31] Altay A., Carter C.B., Rulis P., Ching W.Y., Arslan I., Gülgün M.A. (2010). Characterizing CA2 and CA6 using ELNES. J Solid State Chem..

[32] Coble R.L. (1961). Sintering Crystalline Solids. I. Intermediate and Final State Diffusion Models. J. Appl. Phys..

[33] Pillai S.K.C., Baron B., Pomeroy M.J., Hampshire S. (2004). Effect of oxide dopants on densification, microstructure and mechanical properties of alumina-silicon carbide nanocomposite ceramics prepared by pressureless sintering. J. Eur. Ceram. Soc..

[34] Bae S.I., Baik S. (1993). Determination of Critical Concentrations of Silica and/or Calcia for Abnormal Grain Growth in Alumina. J. Am. Ceram. Soc..

[35] Guo J.K., Kou H.M., Li J. (2011). Study on High Temperature Structural Ceramics.

[36] Deng Y.C. (2009). Effects of Adding Eu^3+^, La^3+^ on the Structures and Properties of Alumina Ceramics.

[37] Koyama T., Nishiyama A., Niihara K. (1994). Effect of grain morphology and grain size on the mechanical properties of Al_2_O_3_ ceramics. J. Mater. Sci..

[38] Dogan C., Hawk J. (1995). Effect of grain boundary glass composition and devitrification on the abrasive wear of Al_2_O_3_. Wear.

[39] Chen C., Chen H., Wang J., Sun B. (2009). Synthesis, Properties and Application of Calcium Hexaluminat. Bull. Chin. Ceram. Soc..

[40] Evans A., Marshall D. (1980). Wear mechanisms in ceramics. Proc. of Int. Conf. on Fundaments of Friction and Wear of Materials.

[41] Kong Y., Gong J. (1988). Friction and Sliding Wear of Structural Ceramics. Bull. Chin. Ceram. Soc..

[42] West G., Perkins J., Lewis M. (2007). The effect of rare earth dopants on grain boundary cohesion in alumina. J. Eur. Ceram. Soc..

[43] Ikuhara Y., Yoshida H., Sakuma T. (2001). Impurity effects on grain boundary strength in structural ceramics. Mater. Sci. Eng. A.

[44] Meng F., Fu Z., Wang W., Zhang Q. (2010). Microstructural evolution of nanocrystalline Al_2_O_3_ sintered at a high heating rate. Ceram. Int..

[45] Powell-Doǧan C.A., Heuer A.H. (1990). Microstructure of 96% Alumina Ceramics: III, Characterization of High-Calcia Boundary Glasses. J. Am. Ceram. Soc..

